# Medium- and high-intensity rTMS reduces psychomotor agitation with distinct neurobiologic mechanisms

**DOI:** 10.1038/s41398-018-0129-3

**Published:** 2018-07-05

**Authors:** Alesha Heath, Daniel R. Lindberg, Kalina Makowiecki, Avalon Gray, Anders J. Asp, Jennifer Rodger, Doo-Sup Choi, Paul E. Croarkin

**Affiliations:** 1 Experimental and Regenerative Neurosciences, Perth, WA Australia; 2 Mayo Clinic Graduate School of Biomedical Sciences, Mayo Clinic College of Medicine and Science, Rochester, MN USA; 3 School of Biological Sciences, University of Western Australia, Perth, WA Australia; 4 Department of Systems Neuroscience, JFB, University of Goettingen, Goettingen, Germany; 5 Perron Insititute for Neurological and Translational Science, Perth, WA Australia; 6 Department of Molecular Pharmacology and Experimental Therapeutics, Mayo Clinic, Rochester, MN USA; 7 Department of Psychiatry and Psychology, Mayo Clinic, Rochester, MN USA

## Abstract

Definitive data are lacking on the mechanism of action and biomarkers of repetitive transcranial magnetic stimulation (rTMS) for the treatment of depression. Low-intensity rTMS (LI-rTMS) has demonstrated utility in preclinical models of rTMS treatments but the effects of LI-rTMS in murine models of depression are unknown. We examined the behavioral and neurobiologic changes in olfactory bulbectomy (OB) mice with medium-intensity rTMS (MI-rTMS) treatment and fluoxetine hydrochloride. We then compared 10-Hz rTMS sessions for 3 min at intensities (measured at the cortical surface) of 4 mT (LI-rTMS), 50 mT (medium-intensity rTMS [MI-rTMS]), or 1 T (high-intensity rTMS [HI-rTMS]) 5 days per week over 4 weeks in an OB model of agitated depression. Behavioral effects were assessed with forced swim test; neurobiologic effects were assessed with brain levels of 5-hydroxytryptamine, brain-derived neurotrophic factor (BDNF), and neurogenesis. Peripheral metabolomic changes induced by OB and rTMS were monitored through enzyme-linked immunosorbent assay and ultrapressure liquid chromatography-driven targeted metabolomics evaluated with ingenuity pathway analysis (IPA). MI-rTMS and HI-rTMS attenuated psychomotor agitation but only MI-rTMS increased BDNF and neurogenesis levels. HI-rTMS normalized the plasma concentration of α-amino-*n*-butyric acid and 3-methylhistidine. IPA revealed significant changes in glutamine processing and glutamate signaling in the OB model and following MI-rTMS and HI-rTMS treatment. The present findings suggest that MI-rTMS and HI-rTMS induce differential neurobiologic changes in a mouse model of agitated depression. Further, α-amino-*n*-butyric acid and 3-methylhistidine may have utility as biomarkers to objectively monitor the response to rTMS treatment of depression.

## Introduction

Repetitive transcranial magnetic stimulation (rTMS) has been used clinically since 2008 for treatment-resistant major depressive disorder. The virtually infinite parameter space of dosing rTMS (e.g., coil geometry, coil position, focality, intensity, frequency, session length, session number, and brain state) magnifies the challenges of elucidating mechanisms of action and contributes to diverse clinical outcomes. Notwithstanding their limitations, animal and preclinical models are key tools in refining the mechanistic understanding and optimal stimulus dosing approach for rTMS in major depressive disorder. In many rodent models, the focality of human rTMS cannot be appropriately reproduced because even the smallest animal coil stimulates a large portion of the brain^1^. However, coils that achieve focality but at a lower intensity enable the mechanistic study of low-intensity rTMS (LI-rTMS). Low-intensity rTMS may modulate cortical excitability in the frontal cortex of humans, thereby addressing clinical psychiatric symptoms. Furthermore, preclinical LI-rTMS protocols may model the perifocal effects of standard clinical rTMS^2–5^.

The present study examined a mouse olfactory bulbectomy (OB) model to investigate differential effects of rTMS stimulation intensities on depression-related behaviors and neurobiologic characteristics. The OB model was selected because removal of the olfactory bulbs results in modulation of downstream connections with such limbic structures as hippocampus, amygdala, habenular nuclei, and raphe dorsalis, inducing reproducible behavioral changes that are associated with depression^6,7^. In addition, aspects of these behavioral changes, including hyperactivity or psychomotor agitation in the forced swim test (FST)^8–10^, anhedonia^11,12^, neurocognitive impairment^13^, and anxiety^14^ reflect the symptoms of agitated depression in humans, which often shows poor responsiveness to pharmacologic treatment^15–18^. We investigated brain markers of plasticity (i.e., brain-derived neurotrophic factor [BDNF] and hippocampal neurogenesis), brain serotonin, and plasma metabolites in an attempt to better understand how the cellular and molecular effects of rTMS intensity relate to behavioral outcomes.

The study design was motivated by an unmet need for preclinical dose-finding studies of rTMS for treatment-refractory phenotypes and special clinical populations. First, we examined the behavioral and neurobiologic changes in OB mice with medium-intensity rTMS (MI-rTMS) treatment and fluoxetine hydrochloride. We then compared the same outcomes following three different intensities of rTMS to gain insight into relevant neurobiologic mechanisms among variable intensities. Finally, we extended our results by analyzing serum samples for potential biomarkers of depression-like behavior in the OB model and recovery induced by rTMS. We hypothesized that high-intensity rTMS would have the greatest behavioral and neurobiologic impact on an OB model of an agitated, treatment-refractory depression.

## Materials and methods

### Study overview

To examine behavioral (FST) and frontal cortex 5-hydroxytryptamine (5HT) changes, we first compared mice treated with rTMS, fluoxetine, and their respective controls. These five groups included mice undergoing a sham surgery and receiving sham rTMS (*n* = 11), mice undergoing OB and sham rTMS (*n* = 9), mice undergoing OB and MI-rTMS (*n* = 11), mice undergoing OB treated with fluoxetine (*n* = 9), and mice undergoing OB treated with a vehicle (*n* = 9). 5HT changes were measured in subsets of these mice. In a second set of experiments, mice undergoing a sham surgery and sham rTMS (*n* = 11), OB mice treated with sham rTMS (*n* = 12), OB mice treated with LI-rTMS (*n* = 16), OB mice treated with MI-rTMS (*n* = 13), and OB mice treated with HI-rTMS (*n* = 15) had FST testing. Frontal cortex and hippocampal BDNF, hippocampal neurogenesis, and plasma metabolomic studies were conducted in subsets of these treated mice. Table 1 provides an overview of sample sizes. All post-mortem tests and video analyses were blinded.

**Table 1:** Overview of experiment 1 and 2 sample sizes

Experiment 1	FST	5HT
SHAM/SHAM	*n* = 11	*n* = 5
OB/SHAM	*n* = 9	*n* = 3
OB/MI-rTMS	*n* = 11	*n* = 5
OB/Flu	*n* = 9	*n* = 4
OB/Veh	*n* = 9	*n* = 4

Experiment 2	FST	BDNF	Neurogenesis	Metabolomics
SHAM/SHAM	*n* = 11	*n* = 5	*n* = 4	*n* = 4
OB/SHAM	*n* = 12	*n* = 8	*n* = 4	*n* = 9
OB/LI-rTMS	*n* = 16	*n* = 7	*n* = 7	*n* = 11
OB/MI-rTMS	*n* = 13	*n* = 8	*n* = 5	*n* = 10
OB/HI-rTMS	*n* = 15	*n* = 7	*n* = 6	*n* = 9

*BDNF* brain-derived neurotrophic factor, *5HT* 5-hydroxytryptamine, *Flu* fluoxetine, *FST* forced swim test, *HI-rTMS* high-intensity repetitive transcranial magnetic stimulation, *LI-rTMS* low-intensity repetitive transcranial magnetic stimulation, *MI-rTMS* medium-intensity repetitive transcranial magnetic stimulation, *OB* olfactory bulbectomy, *Veh* vehicle

### Study animals

**Fig. 1:**
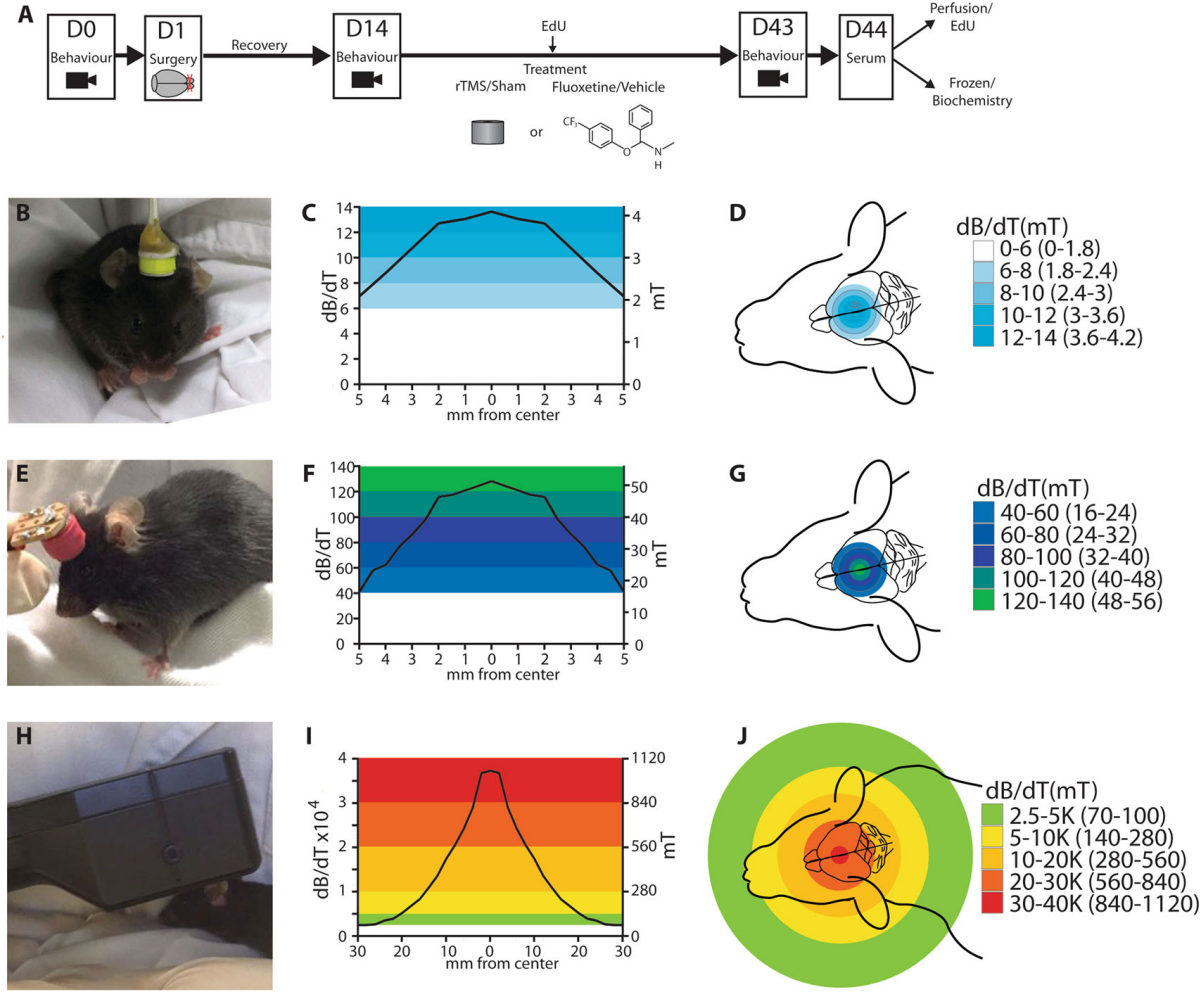
Overview of experimental design. **a** Study design showing timeline and control groups. Study design showing timeline and control groups. Mice received olfactory bulbectomy (OB) or a sham procedure (surgery with olfactory bulbs left intact). Following a 2-week recovery, Mice that had OB underwent 3-min sessions of 10-Hz LI-rTMS (**b**–**d**), MI-rTMS (**e**–**g**), or HI-rTMS (**h**–**j**) 5 days per week over 4 weeks, or received fluoxetine daily (18 mg/kg in cookie dough). Treatment lasted 4 weeks, and some animals received an injection of EdU at the midpoint. Mice underwent behavioral testing before OB, before treatment, and after treatment. Mice were killed humanely at 24 h after the last treatment, and post-mortem serum samples were collected immediately. Brains were prepared for enzyme-linked immunosorbent assay (ELISA) or EdU histologic evaluation. Behavioral effects were assessed with the forced swim test. Neurobiologic effects were assessed with brain levels of 5-hydroxytryptamine, brain-derived neurotrophic factor, and neurogenesis. Peripheral metabolomic changes induced by OB and rTMS were monitored using ELISA and targeted metabolomics driven by ultrapressure liquid chromatography that was evaluated with ingenuity pathway analysis. D day; EdU 5-ethynyl-2′-deoxyuridine (Thermo Fisher Scientific), rTMS repetitive transcranial magnetic stimulation. Measured magnetic field intensity profiles (in d*B*/d*T* and mT) at 2 mm beneath the coil surface (equivalent to cortical surface) for coils used to deliver **d** low-intensity (LI-rTMS)**, g** medium-intensity (MI-rTMS), and **j** high-intensity (HI-rTMS). Additional information about magnetic field intensity parameters is provided in supplementary information

C57BL/6J mice (male sex, aged 8 weeks at start of experiment) were group-housed and maintained under a standard 24-h light-dark cycle (e.g., lights on 0600 and lights off 1800) with ad libitum access to food and water. Each cage contained up to five animals. Mice with aggressive behaviors after OB were moved to separate housing. Housing and bullying were taken into account in statistical analyses. All procedures were approved by the University of Western Australia animal ethics committee (RA03/100/1298) and conformed to US National Institutes of Health guidelines. Experimental design and timeline are shown in Fig. 1a.

### Olfactory bulbectomy

OB was performed according to several previously established surgical protocols^14,19–21^. Briefly, mice were anesthetized with intraperitoneal injection of ketamine (75 mcg/kg; Troy Laboratories Pty Ltd, USA) and medetomidine (1 mg/kg; Troy Laboratories Pty Ltd, USA). A midline incision was made in the skin overlying the skull, and the skin was retracted to reveal the bregma and skull overlying the anterior cranial fossa. Bilateral burr holes 2 mm in diameter were drilled in the skull 4 mm rostral and 2 mm lateral to bregma. The olfactory bulbs were aspirated, and small pieces of hemostatic sponge were used to stop the bleeding before the skin was sutured. Animals receiving a sham operation were treated identically, but the olfactory bulbs were left intact. Following surgery, animals were returned to their home cage. All animals had a 2-week recovery before starting fluoxetine hydrochloride or rTMS treatment. During the second week, all mice were handled daily for 10 min to habituate the animals to the handling associated with rTMS treatment.

### Repetitive transcranial magnetic stimulation

rTMS was delivered daily on weekdays for 3 minutes, to deliver 1800 pulses per session, for 4 weeks (Fig. 1a–j). Detailed specifications of the custom coils and stimulators have been published previously^22,23^ and parameters are described briefly herein. For LI-rTMS, a custom-design copper coil (diameter, 8 mm) was connected to a modified E-cell (Global Energy Medicine, Australia). Magnetic field intensity at the coil base was ~12 mT^22^, and at the cortical surface was 4 mT. For MI-rTMS, a custom-designed air core copper coil (diameter, 8 mm) was controlled by a waveform generator (335141B; Agilent Technologies, USA) connected to a bipolar programmable power supply (BOP 100-4 M; Kepco Inc, USA). Magnetic field intensity at the coil base was approximately 90 mT^23^ and at the cortical surface was 50 mT. For HI-rTMS, a commercially available rat coil (Cool-40 Rat Coil; MagVenture A/S, Denmark) with a 40-mm diameter was used, powered by a MagPro R30 stimulator (MagVenture A/S, Denmark) set to 20% of capable output (Magnetic field intensity coil surface: 1.2 T; cortical surface: 1.0 T). The peak magnetic field adjacent to the coil was measured with a Hall-effect probe (SS94A2D; Honeywell International Inc, USA). Sham-treated animals were handled as if receiving treatment, but the stimulator was not turned on. During treatment, the coil was placed in an anterior position between the ears, overlying the anterior cranial fossa to reliably target the frontal cortex bilaterally (Fig. 1b, e, h). Magnetic field measurements (in d*B*/d*T* and mT) for each coil are shown in Fig. 1. A discussion of the magnetic field intensities used in relation to human rTMS parameters is provided in supplemental information (Supplemental Text and Table 1).

### Fluoxetine treatment

Fluoxetine (Sigma-Aldrich) 18 mg/kg was administered daily for 28 consecutive days, delivered in portioned balls of cookie dough^24^. Mice were monitored individually to ensure they had eaten the full quantity. Control mice (called vehicle group) received the same handling and were given a ball of cookie dough without fluoxetine.

### Ingenuity pathway analysis

Metabolites identified by targeted metabolomics were uploaded into ingenuity pathway analysis (IPA; Qiagen, Redwood City, CA) for stratification and categorization of direct and indirect network interactions using IPA’s functional analysis algorithm and curated IPA ingenuity knowledge base (IPAIKB). IPA is a web-based software application that allows for the analysis, integration, and interpretation of omics datasets by utilizing known molecular and genetic pathways and established relationships with cellular processes and metabolites. We utilized this integrative database in order to identify pathways and processes affected by bulbectomy and multiple rTMS parameters. Prior to entry into IPA, each dataset of identified metabolites was sorted by Chemical Abstract Service (CAS) number. A metabolomics analysis was carried out using default IPA settings, excluding pathways specific to cancer cell lines. To minimize the incidence of false positive results, expression value threshold filters were set to a 1.25 fold-change ratio between bulbectomized and sham-operated animals with a minimum corrected confidence value of *P* ≤ 0.05. The threshold filter for analysis of rTMS treatment was set at 1.5 fold-change ratio between treated and untreated groups. Using these criteria, IPA was able to generate a reference dataset consisting of all significant and non-significant metabolites identified in sham-treated animals and animals treated with various intensities of rTMS, as well as a focus set of metabolites consisting of those present in significantly different levels than the reference dataset. Biological functions, disease states, and canonical pathways associated with our reference and focus set of metabolites were generated by IPA. The IPA functional analysis generated statistical significances derived from the association of our focus metabolite datasets with molecules already established with biological processes and canonical pathways using a right-tailed Fisher’s exact test, where *P* ≤ 0.05 was considered significant.

### Data analysis

Statistical analysis and graphing were carried out using GraphPad Prism 5 (GraphPad Software Inc., La Jolla, CA). Behavioral measures, brain 5HT and BDNF levels, and the number of proliferating cells were analyzed using ANOVA or MANOVA with Sidak post hoc or Dunnett’s T. For forced swim test data, we analyzed treatment effects within-subjects, expressed as the difference between post-surgery and post-treatment scores. All data met the homogeneity of error variance assumption, except for BDNF frontal cortex data, which were log transformed and then met assumptions.

The intensity-dependent effects of rTMS on plasma metabolites were analyzed by one-way ANOVA with Tukey’s post-hoc analysis. IPA was carried out by averaging the metabolite concentrations under each treatment parameter and comparing these mean values to the average metabolite concentration of the relevant control group in order to calculate the fold-change in metabolite concentration. We also investigated possible correlations between FST performance and BDNF levels, neurogenesis and metabolite levels. The threshold for statistical significance for all experiments was set at *P* < 0.05 except where corrected *P*-values were used and these cases are indicated in the text.

Power calculations from the first set of behavioral (FST) data including MI-rTMS and fluoxetine demonstrated that *n* = 4 (per group) would provide 0.8 power to detect a 15% differences (*P* < 0.05) in a one-way ANOVA with four pairwise comparisons, with two-sided equality.

All animals underwent behavioral testing and were then randomly allocated to a particular type of post-mortem test. We assumed that the post-mortem tests would be less variable than behavioral tests, so animals were allocated to various tests to ensure that each group size was a minimum of *n* = 4. The variation in group size was due to our efforts to reconcile randomizing animals from different surgery sessions, technical and timing requirements of experiments, a small number of experimental failures, and attempting to keep group size within experiments as similar as possible. Only one group (OB-Sham for 5HT ELISA) was smaller than 4, but that dataset had low variability, and a subsequent power analysis confirmed that the sample size provided 0.9 power.

### Supplemental Information

The Supplemental Appendix describes an estimate of motor thresholds for each rTMS intensity, 5-ethynyl-2′-deoxyuridine (EdU; Thermo Fisher Scientific) labeling of newly born cells, behavioral analysis, and brain and serum collection. The Supplemental Appendix also summarizes serotonin and BDNF enzyme-linked immunosorbent assays, analysis of neurogenesis, and metabolomics.

## Results

### Tolerability

rTMS at all intensities was well tolerated by the mice and there was no sign that the animals felt any sensations during the stimulation and no seizures, freezing, or avoidance behaviors were noted for any of the intensities applied. Furthermore, there were no signs of ongoing pain or discomfort following the stimulation as determined by the facial grimace score^25^.

### Reduction of hyperactivity in the FST by MI-rTMS but not fluoxetine is independent of serotonin levels

**Fig. 2:**
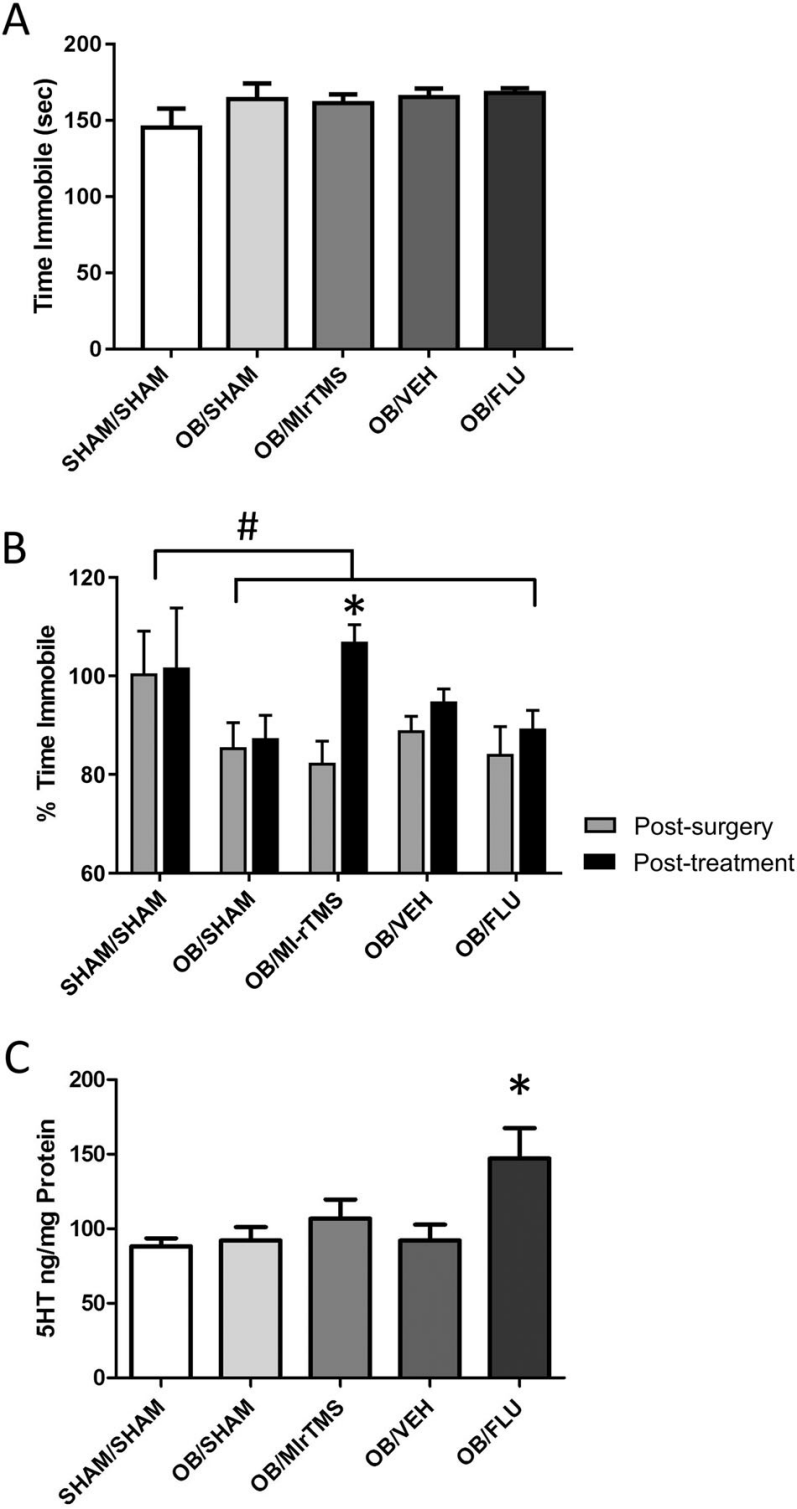
MI-rTMS and flu effects in mice with OB. **a** Time (in seconds) spent immobile at baseline (pre-surgery) for all groups, confirming that there were no differences between animals at the start of the experiment. **b** Time spent immobile, expressed as a percentage of pre-surgery values of mice with intact olfactory bulbs (sham/sham) and mice with OB treated with MI-rTMS or Flu. For all groups, OB resulted in a significant decrease in the time spent immobile, representing hyperactivity. Behavior was significantly improved by 4 weeks of treatment with MI-rTMS but not Flu. # indicates that post-surgery levels are significantly different than for untreated animals. * indicates significant differences between post-surgery and post-treatment within a group. **c** Concentration of 5HT in the frontal cortex of sham/sham mice and mice following OB surgery treated with MI-rTMS or Flu. OB alone had no effect on 5HT levels. * indicates that only Flu increased 5HT levels following the OB procedure. Error bars represent standard error of the mean. 5HT 5-hydroxytryptamine, Flu fluoxetine hydrochloride, MI-rTMS medium-intensity repetitive transcranial magnetic stimulation, OB olfactory bulbectomy, Veh vehicle (i.e., control)

As previously reported^8–10^, the FST revealed a significant and reliable decrease in immobility after OB surgery, providing an opportunity to compare the efficacy of rTMS and fluoxetine in restoring normal activity levels. Activity in the FST was not significantly different between groups at baseline (pre-intervention; Fig. 2a) but significantly differed in the change induced by treatment over time (analysis of variance [ANOVA], significant time × group interaction with Greenhouse-Geisser correction for sphericity violation, F(7.06, 164.91) = 3.702 (*P* = 0.001)). Follow-up tests for simple effects of time within each group showed a significant decrease in immobility time in the FST from pre-surgery to post-surgery in all groups (*P*-values < 0.05) except for sham/sham (*P* = 0.42), confirming that OB-induced hyperactivity consistent with a phenotype of agitated depression (Fig. 2b).

To compare the efficacy of MI-rTMS and fluoxetine in reducing OB-induced hyperactivity, we performed further follow-up analyses (Dunnett test), examining rTMS and fluoxetine groups compared with their respective controls and with sham/sham. The MI-rTMS group showed significantly greater improvements between the post-surgery to post-treatment time points in the FST than both OB/sham (Dunnett test, *P* = 0.03) and sham/sham (Dunnett test, *P* = 0.02) (Fig. 2b). However, no significant difference was found between post-surgery and post-treatment for fluoxetine or vehicle groups (Dunnett test, *P* > 0.05) (Fig. 2b). We then investigated whether the behavioral changes were accompanied by a change in 5-hydroxytryptamine (5HT) levels in the frontal cortex at the end of treatment. A significant difference was observed in frontal cortex 5HT levels between groups (ANOVA, F[4, 38] = 4.113; *P* = 0.007) (Fig. 2c). Post hoc tests showed that MI-rTMS did not significantly affect 5HT, but the OB/fluoxetine group had significantly increased frontal cortex 5HT levels compared with both vehicle-treated control (Sidak, *P* = 0.03) and sham/sham (*P* = 0.004) (Fig. 2c). Interestingly, the two control groups (OB/vehicle and sham/sham) were not significantly different from each other, suggesting that OB itself did not alter baseline 5HT concentration (*P* = 0.86).

### Intensity of rTMS differentially reduces hyperactivity in the FST

**Fig. 3:**
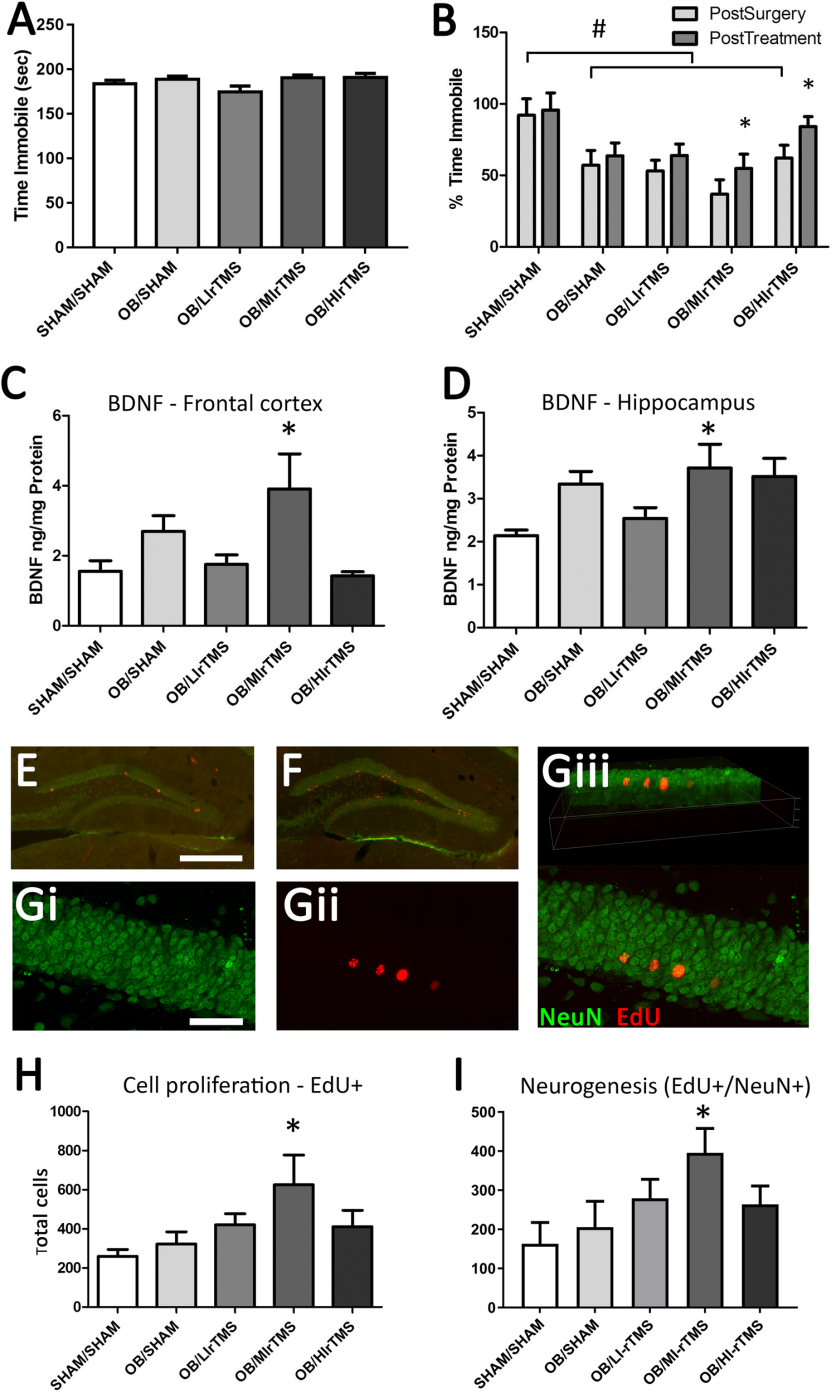
Comparison of rTMS intensities. **a** Time (in seconds) spent immobile at baseline (pre-surgery) for all groups, confirming that there were no differences between animals at the start of the experiment. **b** Time spent immobile in the forced swim test as a percentage of the pre-surgery value for each animal. OB surgery significantly reduced the time spent immobile compared with pre-surgery values, indicating hyperactivity. Behavior was partially rescued by MI-rTMS treatment. # indicates that post-surgery levels are significantly different from untreated animals. * shows significant differences between post-surgery and post-treatment within a group. **c**, **d** BDNF concentration in the frontal cortex (**c**) and hippocampus (**d**). Only MI-rTMS resulted in increased BDNF levels compared with sham/sham treatment in both frontal cortex and hippocampus, but no significant differences were observed between any groups compared with OB/sham. * indicates a significant difference compared with sham/sham. **e**–**g** Immunohistochemistry showing EdU single-labeled and EdU-NeuN double-labeled cells in the granular and subgranular layers of the dentate gyrus. **e**, **f** Low power view of the hippocampus in sham (**e**) and MI-rTMS (**f**)-treated mice showing increased Edu labeling following MI-rTMS. confocal microscopy of MI-rTMS-treated animal confirming co-localization of NeuN (**g**i), EdU (**g**ii), labels merged (**g**iii), with a 3d rotated view showing co-localization of red and green staining within the hippocampal neurons (**g**iii). **h**, **i** Number of EdU-positive cells (**h**) and Edu-NeuN double-labeled cells (**i**) in hippocampus following OB surgery and rTMS treatment. A significant increase was found in the number of newly born neurons following MI-rTMS compared with OB/sham and sham/sham mice. * shows a significant difference compared with sham/sham and OB/sham. Error bars represent standard error of the mean. Scale bars are 500 µm (**e**) and 50 µm (**g**). BDNF brain-derived neurotrophic factor, EdU 5-ethynyl-2′-deoxyuridine (Thermo Fisher Scientific), HI-rTMS high-intensity repetitive transcranial magnetic stimulation, LI-rTMS light-intensity repetitive transcranial magnetic stimulation, MI-rTMS medium-intensity repetitive transcranial magnetic stimulation, NeuN neuron-specific protein, OB olfactory bulbectomy, rTMS repetitive transcranial magnetic stimulation

We then delivered rTMS at different intensities to determine optimal conditions for reducing hyperactive behavior in the FST. We first confirmed that there were no pre-existing differences in the FST behavior prior to intervention (Fig. 3a). A significant change was observed in immobility time in the FST over time and across groups (ANOVA, F[2, 144] = 54.64; *P* < 0.001), indicating that surgery increased activity (agitation) in the FST. All rTMS groups showed some recovery, but only the MI-rTMS (*P* = 0.005) and HI-rTMS (*P* = 0.02) groups improved significantly between post-surgery and post-treatment (Fig. 3b). Confirming our results from the first experiment, none of the rTMS groups showed any change in 5HT levels in the frontal cortex (ANOVA with groups OB-sham, LI-rTMS, MI-rTMS, and HI-rTMS (F[3, 25] = 1.106; *P* = 0.37) (data not shown)).

### Brain-derived neurotrophic factor

Our initial experiment ruled out a role for 5HT in the effects of MI-rTMS and in the rescue of OB-induced behavior. Therefore, we investigated frontal cortical and hippocampal BDNF levels, as well as hippocampal neurogenesis, as possible mechanisms underlying behavioral change in the cohorts of different rTMS intensity.

ANOVA showed a significant main effect of rTMS treatment on BDNF concentrations in the frontal cortex (ANOVA, F[4, 29] = 3.368; *P* = 0.02) and the hippocampus (F[4, 29] = 2.983; *P* = 0.04). We followed up by first comparing all groups to the sham/sham group. In the frontal cortex, only the MI-rTMS group had significantly higher BDNF levels than the sham/sham group (Dunnett test, *P* = 0.048) (Fig. 3c). In the hippocampus, BDNF levels were increased to a similar level in the MI-rTMS, HI-rTMS, and OB-sham groups compared with the sham/sham group, although this increase was significant only for MI-rTMS (Dunnett test, *P* = 0.04) (Fig. 3d). No significant differences in BDNF levels were observed between any rTMS-treated group and the OB-sham group (Dunnett test, *P* > 0.05).

### Hippocampal neurogenesis

In a separate cohort, we analyzed cell genesis by injecting EdU at the midpoint of rTMS treatment. The mice were killed humanely 2 weeks later. We compared the density of newly born neurons (EdU and neuron-specific protein [NeuN] double-labeled cells) following the different rTMS intensities (Fig. 3e–g). A significant difference was detected between groups (ANOVA, F[1, 24] = 2.989; *P* = 0.04), but only MI-rTMS had a significantly higher number of newly born neurons (Fig. 3e, f, h, i) compared with the sham/sham group (Dunnett test, *P* = 0.02). Further follow-up analyses showed that hippocampal neurogenesis in MI-rTMS was also significantly greater than OB-sham (Dunnett test, *P* = 0.02), but no other groups were significantly different to the OB-sham group (Dunnett test, *P* > 0.05) (Fig. 3e, f, h, i). The percentage of newly born neurons positive for NeuN was not significantly different between groups (data not shown).

### Plasma metabolomics

**Fig. 4:**
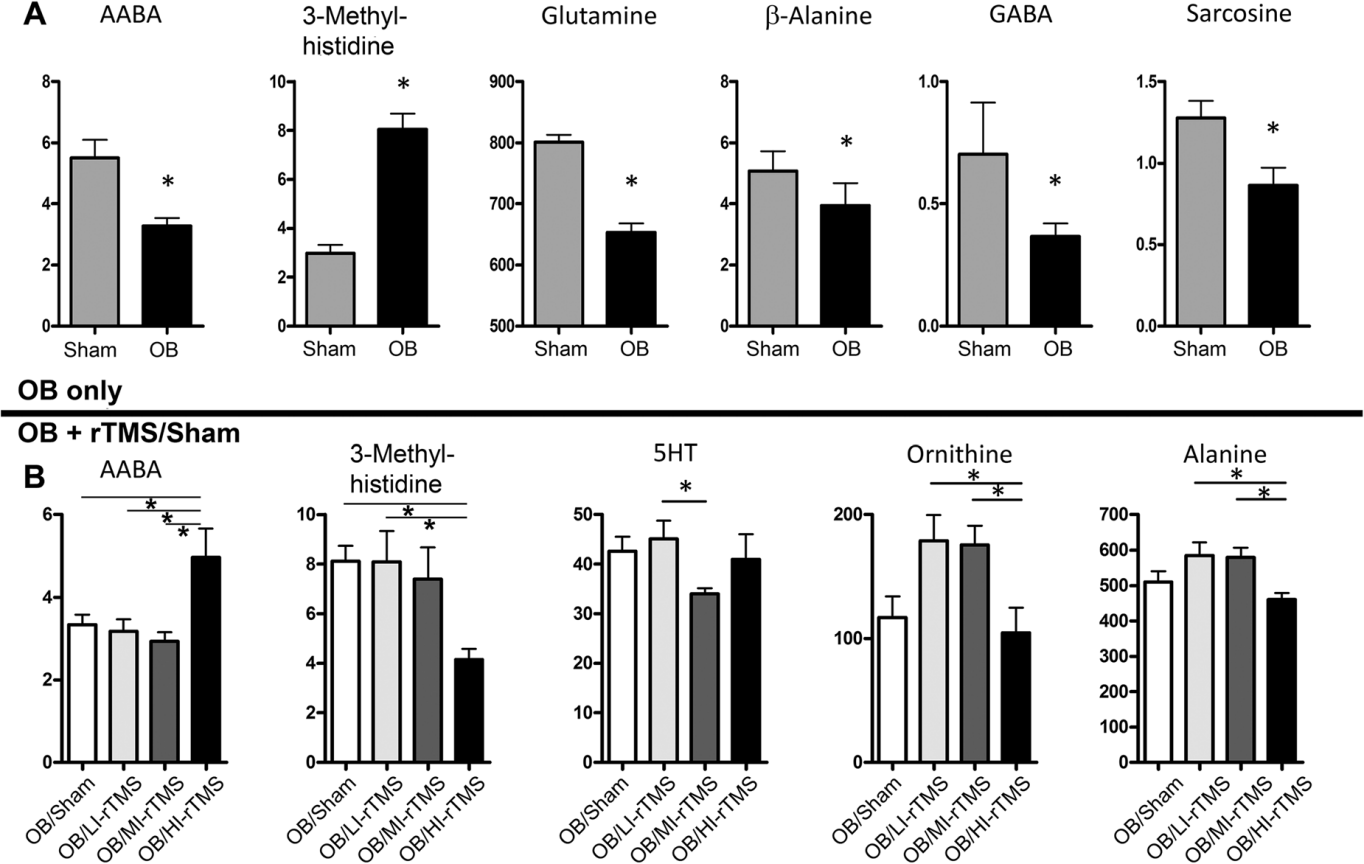
Metabolomics of OB surgery and rTMS exposure. **a** The OB surgery decreased AABA, glutamine, β-alanine, GABA, and sarcosine levels. OB surgery increased 3-methylhistidine. **b** After the OB procedure, rTMS exposure appeared to interact with 7 metabolites, but post hoc analyses showed that only AABA was significantly upregulated and 3-methylhistidine was significantly downregulated with HI-rTMS. However, 5 metabolites—AABA, 3-methylhistidine, 5HT, ornithine, and alanine—were differentially affected by different stimulation intensities. Units are µM. AABA α-aminobutyric acid, 5HT 5-hydrotryptophan, GABA γ-aminobutyric acid, HI-rTMS high-intensity repetitive transcranial magnetic stimulation, LI-rTMS low-intensity repetitive transcranial magnetic stimulation, MI-rTMS medium-intensity repetitive transcranial magnetic stimulation, OB olfactory bulbectomy, rTMS repetitive transcranial magnetic stimulation. *P<0.05

The metabolic effects of OB and the different intensities of rTMS treatment were examined using targeted metabolomics driven by ultrahigh performance liquid chromatography. In total, 6 of the 42 examined metabolites were found to be significantly altered in the plasma of OB mice compared with their sham-treated counterparts. OB decreased the concentration of three bioactive amines—γ-aminobutyric acid (GABA) (*P* = 0.049), AABA (*P* = 0.001), and their molecular precursor glutamine (*P* < 0.001)—as well as β-alanine (*P* = 0.048) and sarcosine (*P* = 0.04). Only 3-methylhistidine was significantly increased by OB (*P* < 0.001) (Fig. 4a). After the OB procedure and rTMS treatment at different intensities, 7 of 42 examined metabolites were determined to significantly interact with rTMS (Supplemental Table 2). However, post hoc analysis revealed that only AABA and 3-methylhistidine were affected by any specific rTMS treatment, being significantly upregulated (*P* = 0.004) and downregulated, respectively, by HI-rTMS (*P* = 0.03) (Fig. 4b). Interestingly, these two metabolites were also significantly altered by OB, and HI-rTMS had the effect of restoring normal levels. All rTMS intensities produced different changes in the plasma metabolic profile, and post hoc analysis revealed that 5 of the 7 metabolites were affected differentially by different stimulation intensities (Fig. 4b).

IPA revealed that the top 4 canonical pathways affected by OB included glutamate-dependent acid resistance (*P* = 0.006), glutamine degradation (*P* = 0.009), glutamine biosynthesis (*P* = 0.02), and GABA receptor signaling (*P* = 0.02). Glutamate receptor signaling was also significantly affected by OB (*P* = 0.02) (Supplemental Fig. 1).

The IPA results were suggestive that various rTMS stimulation parameters had differential effects on peripheral metabolites. For example, LI-rTMS altered metabolites associated with glutamate-dependent acid resistance (*P* = 0.006), inducible nitric oxide synthase signaling (*P* = 0.009), the antiproliferative role of the somatostatin II receptor (*P* = 0.01), inhibition of angiogenesis by thrombospondin-1 (*P* = 0.01), and neuronal nitric oxide synthase signaling in neurons (*P* = 0.01) (Supplemental Fig. 2A). MI-rTMS significantly affected some of the same canonical pathways, such as glutamate-dependent acid resistance (*P* < 0.001) and neuronal nitric oxide synthase signaling in neurons (*P* < 0.001), but also affected arginine biosynthesis (*P* < 0.001), the citrulline metabolism superpathway (*P* < 0.001), and aspartate biosynthesis (*P* < 0.001) (Supplemental Fig. 2B). LI-rTMS and MI-rTMS also modulated canonical pathways associated with glutamate degradation and GABA receptor signaling (Supplemental Figs. 2A and 2B). Similar to other rTMS stimulation paradigms, HI-rTMS significantly altered glutamate-dependent acid resistance (*P* = 0.006) and GABA receptor signaling (*P* = 0.02) (Supplemental Fig. 2C). It also upregulated canonical pathways involved in glutamate degradation (*P* = 0.02), albeit by altering a different branch of the catabolic pathway. HI-rTMS also significantly altered canonical pathways associated with histamine biosynthesis (*P* = 0.006) and ceramide degradation (*P* = 0.02). Notably, glutamate-dependent acid resistance, glutamate degradation, and GABA receptor signaling pathways were downregulated by OB and upregulated by all rTMS intensities.

### Correlations

When all animals were included in the analysis, FST performance post treatment showed a significant negative correlation with BDNF levels (Frontal cortex: Pearson’s correlation −0.365; *P* = 0.034; Hippocampus: Pearson’s correlation −0.359; *P* = 0.037) and a significant positive correlation AABA (Pearson Correlation 0.474; *P* = 0.029), but these correlations were not observed within any individual treatment group. When groups were analyzed separately, only animals in the Sham-OB group showed a positive correlation with 3-methyl histidine (Pearson’s correlation 0.694; *P* = 0.038).

## Discussion

To our knowledge, this is the first study to suggest that MI-rTMS and HI-rTMS improved hyperactivity in mice with OB, while fluoxetine did not. These findings suggest that OB may be a valid murine model of an agitated, treatment-resistant depression. MI-rTMS-treated mice had increased BDNF levels in the frontal cortex and hippocampus, as well as increased neurogenesis in the hippocampus. None of the rTMS-treated groups had increased serotonin levels. Plasma metabolites such as AABA and 3-methylhistidine, which are involved in glutamate and GABA metabolic signaling pathways, may have utility for further translational research of biomarkers for rTMS.

Similar to the OB model used in the present study, some forms of depression in humans are associated with an agitated phenotype marked by increased stress reactivity and psychomotor agitation, increased nocturnal activity, and impaired concentration^18^. Previous studies of OB in mice have consistently revealed a phenotype of psychomotor agitation^7–10^ and we show for the first time that this behavior is resistant to treatment with fluoxetine, similar to clinical human populations with treatment-resistant depression^15–18,26^. Previous studies have demonstrated that development of the agitated phenotype is influenced by strain and the time post-surgery^27,28^. Future efforts should include a comprehensive battery of behavioral tests examining locomotion, activity, and anxiety, as well as circadian rhythm and hedonic behaviors, in response to OB and anti-depressant treatment, to further characterize the phenotype and validity of the model.

Although many studies have investigated the role of rTMS frequency on brain excitability and function, less focus has been given to the role of intensity, perhaps because the mechanisms activated by rTMS have always been assumed to involve traditional forms of synaptic plasticity such as long-term potentiation and long-term depression, which require action potential firing^29^. However, recent work in humans and in animal models has shown that LI-rTMS and MI-rTMS (subthreshold) can have a notable effect on the brain^5,22,30,31^. In our study, both MI-rTMS and HI-rTMS reduced FST hyperactivity in the OB model despite being sub- and at-threshold, respectively, and causing different neurobiologic outcomes and recruiting different mechanisms of action, as described. These results contrast with previous studies showing that rTMS induced hyperactivity in the open field in intact, normal rats^32^ although extensive differences in stimulation parameters, coil geometry, model, and species preclude a useful comparison between these two studies. Nonetheless, reports of rTMS modulating activity in preclinical studies suggest that it will be important to elucidate whether the improvement in FST following MI and HI-rTMS was due to rescue of the OB-induced psychomotor agitation, or a more general effect due to increased psychomotor inhibition.

Our experiments do not allow us to determine which intensity (LI, MI, or HI) provides better outcomes in our model because the behavioral response was similar for both intensities. However, we speculate that MI-rTMS may provide longer lasting anti-depressant effects than HI-rTMS due to the increase in neurogenesis observed only at medium intensity. The lack of effect on neurogenesis of HI-rTMS suggests a transient anti-depressant effect, and is consistent with the occurrence of relapse in human patients once treatment has ceased. It will be important to carry out longitudinal studies to determine when anti-depressant effects are first observed and how long they persist. It would also be interesting to test a two-step treatment protocol: initial delivery of HI-rTMS might provide rapid benefits, which could then be followed with MI-rTMS to integrate these benefits into long-term structural changes through increases in neurogenesis.

Our work confirms previous suggestions that MI-rTMS upregulates BDNF following OB, in various limbic structures including the frontal cortex and hippocampus^12,33^, but the involvement of the protein in the behavioral phenotype is still unknown, just as the relationship of BDNF to depression in humans is still under debate^33–36^. The present data argue against the possibility that increased BDNF level could be a simple consequence of increased locomotor activity following OB^37–39^, because MI-rTMS reduced hyperactivity while increasing BDNF concentration. Rather, BDNF upregulation may be a beneficial response to MI-rTMS with increased neuroplasticity^12^. The increase in BDNF induced by MI-rTMS may have been sufficient to drive neurogenesis in the hippocampus: Likely, BDNF levels and neurogenesis are linked in our study because hippocampal BDNF levels have been causally linked with neurogenesis in mouse models of learning, enrichment, and anxiety^39–42^. Interestingly, MI-rTMS increased the number of newly generated cells detected 3 weeks after the EdU injection but did not selectively increase the proportion of these cells that differentiated into neurons. Future work could examine whether MI-rTMS increases proliferation or simply increases the survival of newly born cells, because this result has implications for understanding the integration of newly born cells into the hippocampal network^43,44^. It will also be interesting to investigate possible cognitive changes following OB and MI-rTMS treatment, because increased neurogenesis is associated with changes in learning and memory^45^.

Our negative neurobiologic outcomes for HI-rTMS contrast with previous studies that showed increases in both BDNF^46,47^ and neurogenesis^48^ following rTMS using a human coil. The different cellular responses to stimulation at similar intensities may be due to differences in the focality of the coils. We used a commercial rat coil (MagVenture) that not only delivers a broader field than our small animal coils (Fig. 1) but also differs in shape from the conventional human coils (such as figure-of-eight coils or large circular coils) used in previous studies^49^. We also did not observe an increase in brain BDNF levels following LI-rTMS, consistent with previous work showing that very low-intensity stimulation may be brain region specific: LI-rTMS upregulates BDNF in the visual cortex, midbrain, and cerebellum but not in the retina^50–52^, and the lack of upregulation of BDNF in the hippocampus is consistent with our previous study showing no change in hippocampal dendritic spines following LI-rTMS^53^. Nonetheless, we cannot rule out a localized upregulation in the dentate gyrus with these intensities, which was not detected in our dissection of the whole hippocampus. Overall, our findings highlight that the range of mechanisms called into play by different rTMS intensities has implications for outcomes in different brain regions that may receive different field strengths simply as a result of being at a distance from the focal point of stimulation^54^.

Our serum analysis identified several metabolites that were altered in response to OB and in response to 1 or more rTMS frequencies. However, the concentrations of only 2 metabolites, AABA and 3-methylhistidine, were increased and decreased, respectively, by OB and restored to baseline levels by HI-rTMS treatment but not by LI-rTMS or MI-rTMS. This finding further highlights that MI-rTMS and HI-rTMS have specific effects on the different brain and peripheral markers of agitated depression modeled in our study, even though they may eventuate in similar behavioral outcomes. Interestingly, AABA has been identified as a potential biomarker for treatment-resistant depression in humans^55^, consistent with the clinical population treated with rTMS^56–58^. Therefore, evidence from animal and human studies suggests that AABA and 3-methylhistidine may serve as biomarkers in humans for phenotyping depression. Further longitudinal work could determine if ABBA and 3-methylhistidine have utility for monitoring treatment response to rTMS or precision medicine approaches to brain stimulation interventions^56–58^.

Our IPA identified glutamate degradation, glutamate-dependent acid resistance, GABA receptor signaling, and 4-aminobutryate degradation as systems that were both downregulated following OB surgery and upregulated by HI-rTMS and, to a lesser extent, MI-rTMS. Consistent with our findings, changes in GABAergic and glutamatergic processes in the brain have been reported following OB^59–62^ and in the brain and blood of depressed patients^63^. Furthermore, rTMS in rodents and in humans can selectively alter GABA and glutamate levels in the brain and blood^64–66^. This result adds to clinical evidence that behaviors associated with depression may be mediated by GABAergic and glutamatergic systems^66–70^.

The present findings and prior work suggest that significant changes occur in glutamate and other amino acids in the blood of depressed patients^70^. However, the implications of this outcome are uncertain because peripheral measures do not necessarily reflect brain activity^71,72^. A possible explanation is that activation of the hypothalamic-pituitary-adrenal axis and the autonomic nervous system impacts peripheral metabolite levels in the blood^73–76^. For example, in rats, activation of the sympathetic nervous system increases the activity of glutamate pyruvate transaminase and glutamateoxaloacetate transaminase in the blood. These enzymes accelerate the conversion of glutamate to 2-ketoglutarate, resulting in a net decrease in plasma glutamate levels. Prior work demonstrates that corticotrophin-releasing hormone and adrenaline reduce blood glutamate concentrations in rats^77^. This explanation is compatible with our pathway analysis, implicating glutamate and GABA pathways in OB and in MI-rTMS and HI-rTMS-induced recovery.

In summary, our work suggests that the OB murine model may have utility as a model for agitative, treatment-resistant depression. Future studies with exhaustive behavioral testing could assist with the validation of this model. Variations in rTMS intensity appear to alter brain and metabolomic effects in the OB mouse model of agitated depression. Further work in this area could assist with the optimization of clinical rTMS delivery. Peripheral metabolomic markers might have utility as biomarkers for the diagnosis of specific forms of depression and in monitoring or modifying rTMS treatment. Further translational studies are warranted.

### Disclaimer

The content of this report is the sole responsibility of the authors and does not necessarily represent the official views of the US Department of Health and Human Services, the National Institutes of Health, the National Institute of Alcohol Abuse and Alcoholism, the National Institute of Mental Health, University of Western Australia, National Health and Medical Research Council Australia, Neurotrauma Research Program of Western Australia, or the Ulm Foundation.

## Electronic supplementary material

Supplmental Material

Supplemental Table 1

Supplemental Table 2

Supplemental Figure 1

Supplemental Figure 2
