# Supplementary material for: Medium- and high-intensity rTMS reduces psychomotor agitation with distinct neurobiologic mechanisms
Source: Transl Psychiatry. 2018 Jul 5;8:126. doi: 10.1038/s41398-018-0129-3 (PMC6033856; doi:10.1038/s41398-018-0129-3)
Supplement: Supplementary file 1 — Supplmental Material [file 41398_2018_129_MOESM1_ESM.pdf]

Medium- and High-Intensity rTMS Reduces Psychomotor Agitation With Distinct Neurobiologic Mechanisms

Supplemental Methods and Materials

Relationship of intensities used in the current study to rTMS intensities used in humans: estimation of motor threshold vs raw magnetic field intensities.

A key question is how the intensities of rTMS applied here in mice can be appropriately translated to humans. Although motor evoked potentials (MEP) are the most commonly used tool to estimate intensities in human treatment, we do not think that MEPs are a useful measure when comparing mouse and humans due to differences in coil parameters.

In order to obtain MEP data from mice, it is necessary to use large coils that deliver high intensity fields (1-2 Tesla) to depolarize cortical neurons. Our small coils are simply not strong enough to elicit a MEP, the current required to induce such high intensity fields would melt the coil. Although it provides a strong enough field, the commercial "rat" coil, does not elicit reliable MEPs even in rats[1] presumably due to the shape of the coil.

When a large coil is used, these coils tend to be larger than the size of the mouse head, resulting in low magnetic induction efficiency. As a result, the intensity used to elicit a MEP in a mouse is not a true reflection of the intensity required to activate the motor cortex. At the moment, it is not possible to establish a direct comparison between mouse and human MEPs due to the technical limitations imposed by the coils.

Nonetheless, we attempted to make some rough comparisons as follows: using the commercial rat coil on an anaesthetized mouse, 43% maximum stimulator output MSO was required to elicit a visible twitch in the mouse forelimb with every stimulation. We reduced the MSO until the twitch just disappeared, avoiding any potential discomfort or sensation to the awake mice in our experiments, which would have confounded our analysis of depression-like behaviors. The MSO was 20% and we calculated the field intensity at the surface of the coil to be 1.2 T, and the field intensity at the surface of the cortex to be 1.0 mT. We called this amount the motor threshold in mice, and have related all other intensities to this value, regardless of coil shape or size.

In translating our findings to humans, we hypothesize that the intensity required to depolarize a mouse neuron will be similar to the intensity required to depolarize a human neuron. We therefore suggest that due to differences in coil size and shape, the best approach is to match the magnetic field intensity, as well as the induced electric field intensity and distribution, at the surface of the cortex in both mice and humans. Our approach also has the advantage that we can take into account the distance between the coil surface and the cortical surface, which is 10 times greater in humans than in mice (Supplemental Table 1).

Edu labelling of newly born cells

At the start of the third week of treatment, 50% of mice in each group were randomly chosen to receive an injection of EdU (50mg/kg)[2] to label dividing cells. The remaining 50% were used for analysis of brain metabolites (see below). Two

cohorts were necessary as it was not possible to collect frozen and perfused tissue from the same animals.

Behavioral Analysis

For the forced swim test, mice were placed in a 2,000 mL glass beaker half-filled with 25 ± 1°C tap water for 6 minutes and were monitored with video recording. The latency to the first immobility was recorded, and the total immobility time during the last 4 minutes was measured by a blinded research assistant. Immobility was defined by lack of translational movement and a paucity of limb and body movements beyond those necessary to maintain balance and buoyancy. Mice were removed from the water and thoroughly dried on a heating pad prior to return to their homecage

Brain and serum collection

Immediately, following the final behavioral test, mice were injected with 0.1ml lethobarb (Virbac) and 500ul of blood was collected into capillary tubes by cardiac puncture and centrifuged at 10,000 g for 5 minutes to isolate an acellular plasma fraction. Mice were then processed either for ELISA assays (frozen tissue) or EdU immunohistochemistry (perfusions).

Serotonin and BDNF ELISA

For ELISA assays, the left frontal cortex and hippocampus were homogenized in buffer containing 100 mM PIPES H 7, 500 mM NaCl, 0.2% Triton X-100, 2mM EDTA and mini protease inhibitor tablets (Roche Biochemicals, Indiana USA; 1 per 50ml buffer; (Szapacs et al. 2004). Samples were centrifuged at 4oC for 1 hour and supernatants stored at -80oC. The total amount of serotonin in the frontal cortex was analysed using a Serotonin EIA kit (Enzo Life Sciences®). Both the

frontal cortex and hippocampus homogenates were analysed using ChemiKine BDNF Sandwich Elisa Kit (Millipore). The total amount of protein in each sample was quantified with a BCA protein assay kit (Pierce® BCA Protein Assay Kit, Thermo Fisher Scientific©, Illinois USA). All plates were analysed using a PerkinElmer 2300 spectrophometer with EnSpire software. A standard curve was plotted and fitted with a 4 parameter logistic non-linear curve using the Enspire software. Serotonin and BDNF concentrations (ng/mL) of samples were derived from the standard curve and normalised to the protein concentrations (ng/ml) calculated from each sample.

Analysis of Neurogenesis

Mice were transcardially perfused with 50ml of 4% paraformaldehyde in 0.1 Phosphate Buffer. Brains were postfixed in 4% paraformaldehyde solution at 4oC and transferred to 30% sucrose in PBS 48 hours before cryosectioning into 40μm sections. To identify neurons that had been newly generated during rTMS treatment, sections were stained with mouse anti NeuN (Merck Millipore) and then processed for EdU detection. Sections were blocked and permeabilized with 10% normal donkey serum in 0.1% Triton in 1M PBS then incubated overnight with the anti NeuN (1:400). The following day the sections were incubated with the secondary antibody Donkey anti Mouse (1:600; Life Technologies) and the nuclear dye Hoechst (1:1000; Sigma). Immediately after immunohistochemistry was completed, sections were permeabilized in 0.5% Triton in PBS and washed in 3% BSA in PBS. Sections were incubated in the Click-it EdU reaction cocktail (AF594) for 30 minutes, washed in 3% BSA in PBS. Sections were mounted onto superfrost slides (Menzel Gläser, Lomb Scientific) and cover slipped with Fluoromount (Southern Biotech). Sections were analysed under a Leitz Diaplan fluorescent microscope (Leitz, Wetzlar, Germany) at 100x magnification. All EdU

single labelled and EdU-NeuN double-labelled cells in the granular and sub granular layers of the dentate gyrus were counted. In each section, counts were made in either the left or right hippocampus based on random sampling. Twelve sections were counted in total. For confocal imaging, we used a Nikon confocal C2, NI-E microscope with a 60x oil immersion lens. Sections were imaged using 36 Z-stacks at 1μm thickness and images were collected and maximum projections made using Nis Elements AR software.

Serum analysis: Metabolomics

Amino acids and their metabolites were measured by LCMS as previously described.[3,4] Briefly, plasma samples were spiked with internal standards then deproteinized with cold methanol, followed by centrifugation at 10,000 g for 5 minutes. The supernatant was immediately derivatized with 6-aminoquinolyl-N-hydroxysuccinimidyl carbamate according to the manufacturer instructions of Waters' MassTrak kit. A 10-point calibration standard curve underwent a similar derivatization procedure after the addition of internal standards. Both derivatized standards and samples were analyzed on a triple quadrupole mass spectrometer coupled with an Acquity Ultra Pressure Liquid Chromatography (UPLC) system. Data acquisition was done using a select ion monitor (SRM). Concentrations of 42 analytes of each unknown were calculated against each perspective calibration curve.

Ingenuity Pathway Analysis

Metabolites identified by targeted metabolomics were uploaded into Ingenuity Pathway Analysis (IPA; Qiagen, Redwood City, CA) for stratification and categorization of direct and indirect network interactions using IPA's functional analysis algorithm and curated IPA Ingenuity Knowledge Base (IPAIKB). IPA is a

web-based software application that allows for the analysis, integration, and interpretation of ‘omics data sets by utilizing known molecular and genetic pathways and established relationships with cellular processes and metabolites. We utilized this integrative database in order to predict or identify pathways and processes affected by bulbectomy and multiple rTMS parameters. Prior to entry into IPA, each dataset of identified metabolites was sorted by CAS number. A metabolomics analysis was carried out using default IPA settings, excluding pathways specific to cancer cell lines. To minimize the incidence of false positive results, expression value threshold filters were set to a 1.25 fold-change ratio between bulbectomized and sham-operated animals with a minimum corrected confidence value of $p \leq 0.05$. The threshold filter for analysis of rTMS treatment was set at 1.5 fold-change ratio between treated and untreated groups. Using these criteria, IPA was able to generate a reference data set consisting of all significant and non-significant metabolites identified in sham treated animals and animals treated with various intensities of rTMS, as well as a focus set of metabolites consisting of those present in significantly different levels than the reference data set. Biological functions, disease states, and canonical pathways associated with our reference and focus set of metabolites were generated by IPA. The IPA functional analysis generated statistical significances derived from the association of our focus metabolite data sets with molecules already established with biological processes and canonical pathways using a right-tailed Fisher’s Exact Test, where $p \leq 0.05$ was considered significant (Supplemental Figures 1 and 2 and Supplemental Table 2).

Figure S1. Ingenuity pathway analysis (IPA) revealed that the top four canonical pathways affected by olfactory bulbectomy included glutamate-dependent acid

resistance ($p = 5.93x10^{-3}$), glutamine degradation ($p = 8.89x10^{-3}$), glutamine biosynthesis ($p = 1.77x10^{-2}$), and GABA receptor signaling ($p = 1.77x10^{-2}$). Glutamate receptor signaling was also significantly affected by olfactory bulbectomy ($p = 2.06x10^{-2}$). Physiologically, the metabolic changes resulting from olfactory bulbectomy are associated with increased activation of the brain ($p = 2.8x10^{-3}$), reduced inhibition of presympathetic neurons ($p = 2.97x10^{-3}$), and inhibition of synapse maturation ($p = 2.97x10^{-3}$).

Figure S2. IPA analysis of the metabolomics data suggests that rTMS stimulation parameters exert unique physiological effects. For example, LI-rTMS significantly alters metabolites associated with glutamate-dependent acid resistance ($p = 5.93x10^{-3}$), iNOS signaling ($p = 8.89x10^{-3}$), the antiproliferative role of the somatostatin II receptor ($p = 1.18x10^{-2}$), inhibition of angiogenesis by TSP1 ($p = 1.48x10^{-2}$), and nNOS signaling in neurons ($p = 1.48x10^{-2}$) **(Figure S2A)**. Mid-intensity rTMS significantly affects some of the same canonical pathways such as glutamate-dependent acid resistance ($p = 1.65x10^{-5}$) and nNOS signaling in neurons ($p = 1.64x10^{-4}$), but also affects arginine biosynthesis ($p = 3.92x10^{-5}$), the citrulline metabolism superpathway ($p = 9.61x10^{-5}$), and aspartate biosynthesis ($p = 9.89x10^{-5}$) **(Figure S2B)**. Interestingly, both low-intensity rTMS and mid-intensity rTMS also significantly affect canonical pathways associated with glutamate degradation and GABA receptor signaling **(Figures S2A and B)**. Like other rTMS stimulation paradigms, high-intensity rTMS significantly alters glutamate-dependent acid resistance ($p = 5.93x10^{-3}$) and GABA receptor signaling ($p = 1.77x10^{-2}$) **(Figure S2C)**. It also affected canonical pathways involved in glutamate degradation ($p = 2.06x10^{-2}$), albeit by altering a different branch of the catabolic pathway. High intensity rTMS also significantly altered canonical pathways associated with histamine biosynthesis ($p = 5.93x10^{-3}$) and ceramide degradation ($p = 2.06x10^{-2}$). Perhaps most importantly, many of the pathways that were downregulated by olfactory bulbectomy, including glutamate-dependent acid resistance, glutamate degradation, and GABA receptor signaling, were upregulated by all forms rTMS. Furthermore, while olfactory bulbectomy appears to largely inhibit many metabolic functions, rTMS appears to exert stimulatory effects of multiple pathways, increasing the concentration of metabolites involved in multiple distinct pathways.

Supplemental References

1. Wyckhuys T, De Geeter N, Crevecoeur G, Stroobants S, Staelens S. Quantifying the effect of repetitive transcranial magnetic stimulation in the rat brain by muSPECT CBF scans. *Brain Stimul* 2013;6:554-562.

2. Chehrehasa F, Meedeniya AC, Dwyer P, Abrahamsen G, Mackay-Sim A. EdU, a new thymidine analogue for labelling proliferating cells in the nervous system. *Journal of neuroscience methods.* 2009;177(1):122-130.

3. Nam HW, Karpyak VM, Hinton DJ, et al. Elevated baseline serum glutamate as a pharmacometabolomic biomarker for acamprosate treatment outcome in alcohol-dependent subjects. *Translational psychiatry.* 2015;5:e621.

4. Hinton DJ, Vazquez MS, Geske JR, et al. Metabolomics biomarkers to predict acamprosate treatment response in alcohol-dependent subjects. *Scientific reports.* 2017;7(1):2496.
