# Supplementary material for: Medium- and high-intensity rTMS reduces psychomotor agitation with distinct neurobiologic mechanisms
Source: Transl Psychiatry. 2018 Jul 5;8:126. doi: 10.1038/s41398-018-0129-3 (PMC6033856; doi:10.1038/s41398-018-0129-3)
Supplement: Supplementary file 2 — Supplemental Table 1 [file 41398_2018_129_MOESM2_ESM.pdf]

Table S1. Animal and Human Coil Comparisons

Coil	Intensity at coil surface in mT	Intensity at cortical surface (2 mm distance from base of coil) in mT	% MSO (Magventure rat coil only)	% MT (estimated)
HI-rTMS coil (Rat coil)	1200	1000 (37,312)	20	100
MI-rTMS coil	90	51 (128)	NA	5
LI-rTMS coil	12	4 (14)	NA	0.4

Abbreviations: HI-rTMS, high-intensity repetitive transcranial magnetic stimulation; LI-rTMS, low-intensity repetitive transcranial magnetic stimulation; MI-rTMS, medium-intensity repetitive transcranial magnetic stimulation; mm, Millimeter; mT, Millitesla ; %MSO, percentage of maximum stimulator output; %MT, percentage of motor threshold.
