# Supplementary material for: Medium- and high-intensity rTMS reduces psychomotor agitation with distinct neurobiologic mechanisms
Source: Transl Psychiatry. 2018 Jul 5;8:126. doi: 10.1038/s41398-018-0129-3 (PMC6033856; doi:10.1038/s41398-018-0129-3)
Supplement: Supplementary file 3 — Supplemental Table 2 [file 41398_2018_129_MOESM3_ESM.pdf]

Table S2. Plasma Concentrations of Targeted Metabolomics Among the 4 Group Comparisons

	Plasma Concentration, Mean (SD), µM					
**Metabolite[a]**	**OB/Sham**	**OB/LI-rTMS**	**OB/MI-rTMS**	**OB/HI-rTMS**	**rTMS Effect?**	***P* Value**
Histidine	62.79 (7.83)	79.92 (10.50)	83.90 (10.14)	88.97 (10.81)	No	.33
Hydroxyproline	16.68 (1.83)	16.72 (1.08)	16.81 (1.35)	15.75 (0.88)	No	.94
1-Methylhistidine	2.80 (0.51)	3.55 (0.64)	4.01 (0.67)	2.67 (0.65)	No	.34
**3-Methylhistidine**	**8.10 (0.63)**	**8.04 (1.23)**	**7.41 (1.29)**	**4.03 (0.43)**	**Yes**	**.03**
Asparagine	34.98 (1.99)	41.31 (4.18)	41.93 (3.93)	42.57 (3.51)	No	.46
Phosphoethanolamine	3.26 (0.71)	2.07 (0.51)	3.19 (0.77)	4.68 (1.05)	No	.13
**Arginine**	**78.89 (14.37)**	**45.57 (14.18)**	**49.31 (16.59)**	**101.50 (13.52)**	**Yes**	**.04**
Carnosine	3.85 (0.70)	3.70 (0.45)	5.32 (1.56)	4.19 (0.82)	No	.63
Taurine	770.80 (67.50)	755.20 (40.55)	944.0 (104.30)	729.70 (75.76)	No	.18
Anserine	6.43 (1.31)	5.97 (1.02)	8.95 (2.79)	6.10 (1.57)	No	.61
Serine	128.70 (9.97)	143.50 (10.94)	145.90 (9.55)	133.30 (7.10)	No	.56
Glutamine	652.60 (15.25)	714.10 (25.27)	722.20 (33.42)	750.30 (31.82)	No	.12
Ethanolamine	28.82 (1.87)	30.33 (2.41)	33.31 (2.67)	28.32 (3.99)	No	.60
Glycine	335.80 (15.91)	364.80 (17.79)	370.20 (29.40)	339.60 (23.63)	No	.62
Aspartic acid	3.14 (0.37)	2.94 (0.30)	4.69 (1.02)	3.49 (0.51)	No	.19
Sarcosine	0.87 (0.11)	1.22 (0.10)	1.07 (0.20)	1.42 (0.14)	No	.07

Citrulline	61.00 (5.01)	68.61 (3.72)	73.44 (5.94)	63.75 (2.31)	No	.24
Glutamate	81.31 (9.05)	80.79 (5.72)	116.20 (16.73)	82.17 (10.15)	No	.08
β-Alanine	3.08 (0.53)	3.08 (0.32)	4.54 (0.65)	4.10 (0.59)	No	.13
Threonine	110.60 (10.11)	124.80 (7.92)	128.60 (8.44)	105.60 (5.98)	No	.17
**Alanine**	**508.40 (30.31)**	**584.60 (35.87)**	**582.10 (27.52)**	**456.30 (19.09)**	**Yes**	**.01**
GABA	0.37 (0.05)	0.54 (0.12)	0.89 (0.28)	0.84 (0.17)	No	.17
α-Aminoadipic acid	8.09 (0.44)	7.31 (0.50)	8.40 (0.67)	7.11 (0.59)	No	.32
Proline	76.57 (6.30)	104.80 (12.62)	102.30 (8.31)	80.28 (3.01)	No	.06
Hydroxylysine 2	0.69 (0.09)	0.77 (0.04)	0.68 (0.08)	0.69 (0.07)	No	.75
**AABA**	**3.35 (0.24)**	**3.22 (0.28)**	**3.00 (0.21)**	**4.97 (0.69)**	**Yes**	**.005**
**Ornithine**	**116.40 (17.57)**	**177.30 (20.16)**	**177.60 (15.96)**	**103.30 (20.32)**	**Yes**	**.01**
**Cystathionine 1**	**2.25 (0.37)**	**3.01 (0.63)**	**1.37 (0.08)**	**2.97 (0.42)**	**Yes**	**.04**
Lysine	251.70 (13.36)	263.80 (9.97)	260.80 (10.56)	242.80 (18.79)	No	.67
Cystine	1.12 (0.07)	1.07 (0.07)	1.08 (0.08)	1.03 (0.07)	No	.88
Tyrosine	71.94 (5.89)	90.27 (7.93)	85.09 (6.21)	85.86 (4.08)	No	.24
Methionine	40.44 (2.68)	43.91 (1.82)	46.90 (2.12)	40.73 (2.35)	No	.16
Valine	158.00 (14.05)	179.20 (8.88)	181.40 (11.48)	147.90 (13.46)	No	.15
Isoleucine	73.64 (8.95)	81.83 (4.72)	82.26 (5.49)	66.17 (8.14)	No	.31
Allo isoleucine	0.03 (0.02)	0.07 (0.03)	0.15 (0.08)	0.05 (0.02)	No	.34

Leucine	117.10 (11.33)	129.90 (7.69)	131.20 (8.48)	102.80 (13.03)	No	.18
Phenylalanine	60.65 (4.14)	74.19 (4.81)	70.45 (5.16)	68.94 (4.27)	No	.24
Tryptophan	51.52 (2.94)	51.18 (1.57)	54.07 (2.35)	56.72 (2.36)	No	.31
**Serotonin**	**42.58 (2.98)**	**44.94 (3.70)**	**34.21 (1.18)**	**44.31 (2.19)**	**Yes**	**.03**

Abbreviations: AABA, α-amino-n-butyric acid; GABA, γ-aminobutyric acid; HI-rTMS, high-intensity repetitive transcranial magnetic stimulation; LI-rTMS, low-intensity repetitive transcranial magnetic stimulation; MI-rTMS, medium-intensity repetitive transcranial magnetic stimulation; OB, olfactory bulbectomy; rTMS, repetitive transcranial magnetic stimulation.

[a] Bold indicates statistical significance.
