# Supplementary figures and images for: Medium- and high-intensity rTMS reduces psychomotor agitation with distinct neurobiologic mechanisms
Source: Transl Psychiatry. 2018 Jul 5;8:126. doi: 10.1038/s41398-018-0129-3 (PMC6033856; doi:10.1038/s41398-018-0129-3)

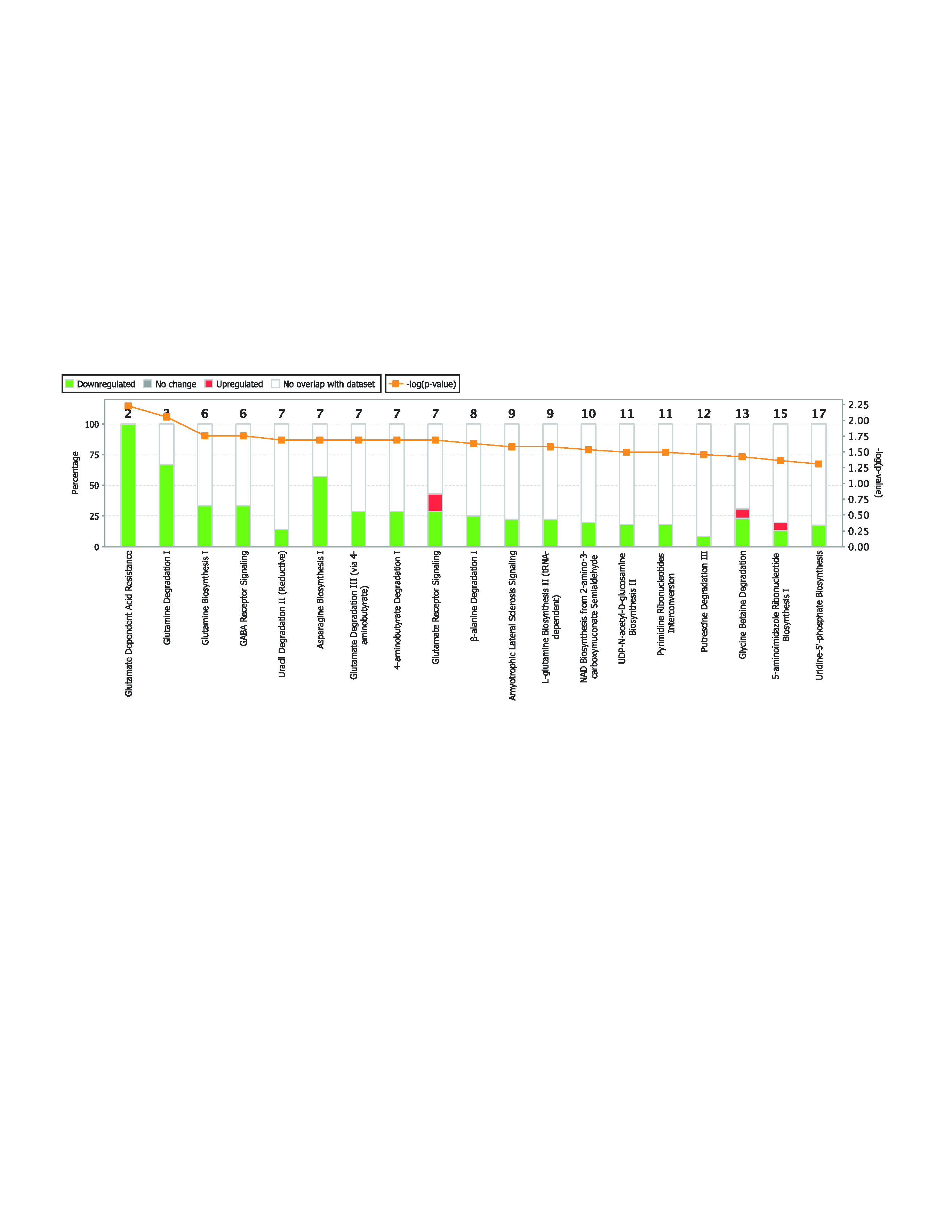

Supplement: Supplementary file 4 — Supplemental Figure 1 [file 41398_2018_129_MOESM4_ESM.tif]

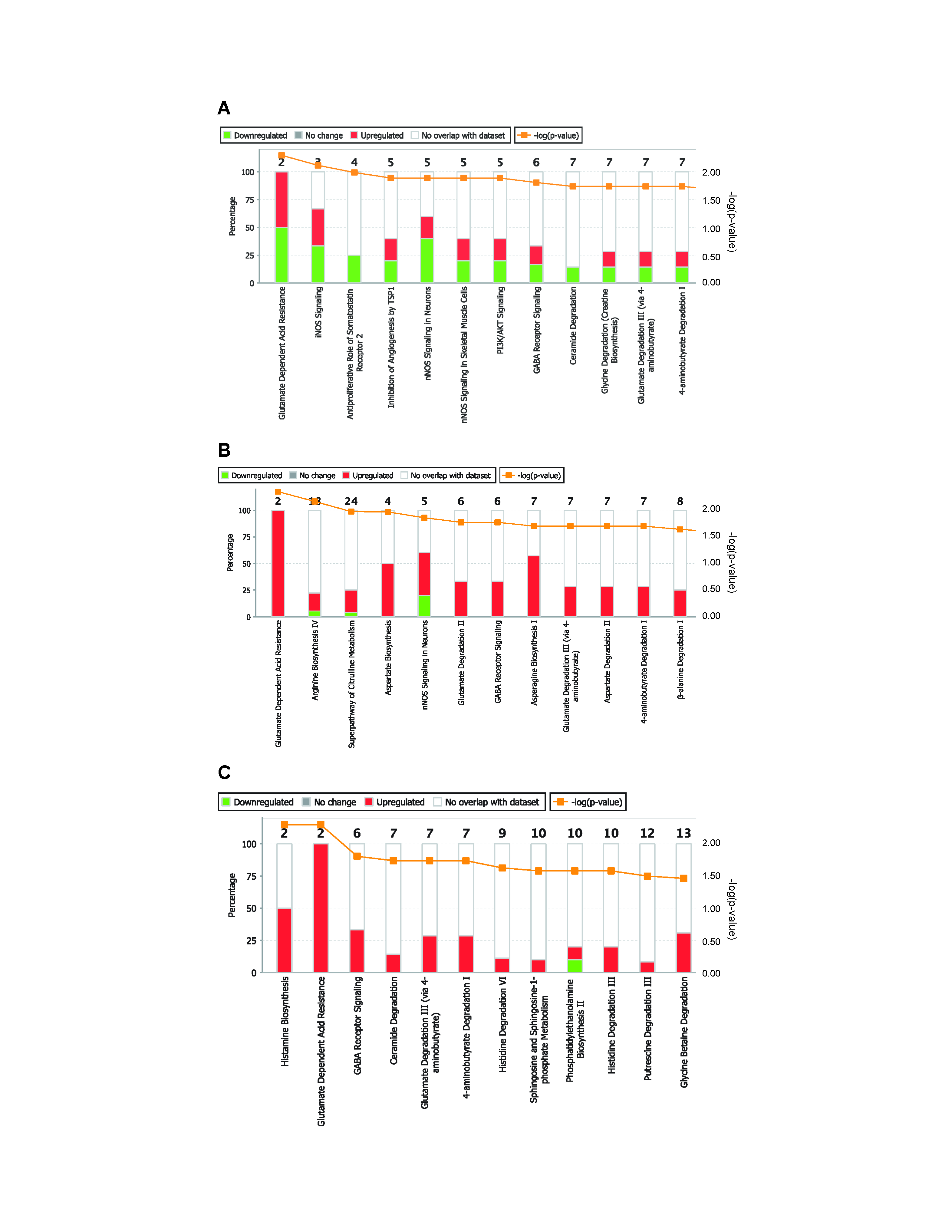

Supplement: Supplementary file 5 — Supplemental Figure 2 [file 41398_2018_129_MOESM5_ESM.tif]
